# Supplementary material for: Predicting visual function by interpreting a neuronal wiring diagram
Source: Nature. 2024 Oct 2;634(8032):113–23. doi: 10.1038/s41586-024-07953-5 (PMC11446822; doi:10.1038/s41586-024-07953-5)
Supplement: Supplementary file 6 — CRF and ERF predictions for individual TmY4 and TmY9 cells. Analogous to Supplementary Data 3, but for TmY target types. Shown are the top four monosynaptic pathways, the strongest pathway passing through each of the top ten intermediary types (ranking from Extended Data Fig. 7), and the trisynaptic pathway Tm1–TmY–Dm3–TmY (see the section entitled Prediction of spatial normalization). [file 41586_2024_7953_MOESM6_ESM.zip › DataS4/TmY9q/720575940613299882.pdf]

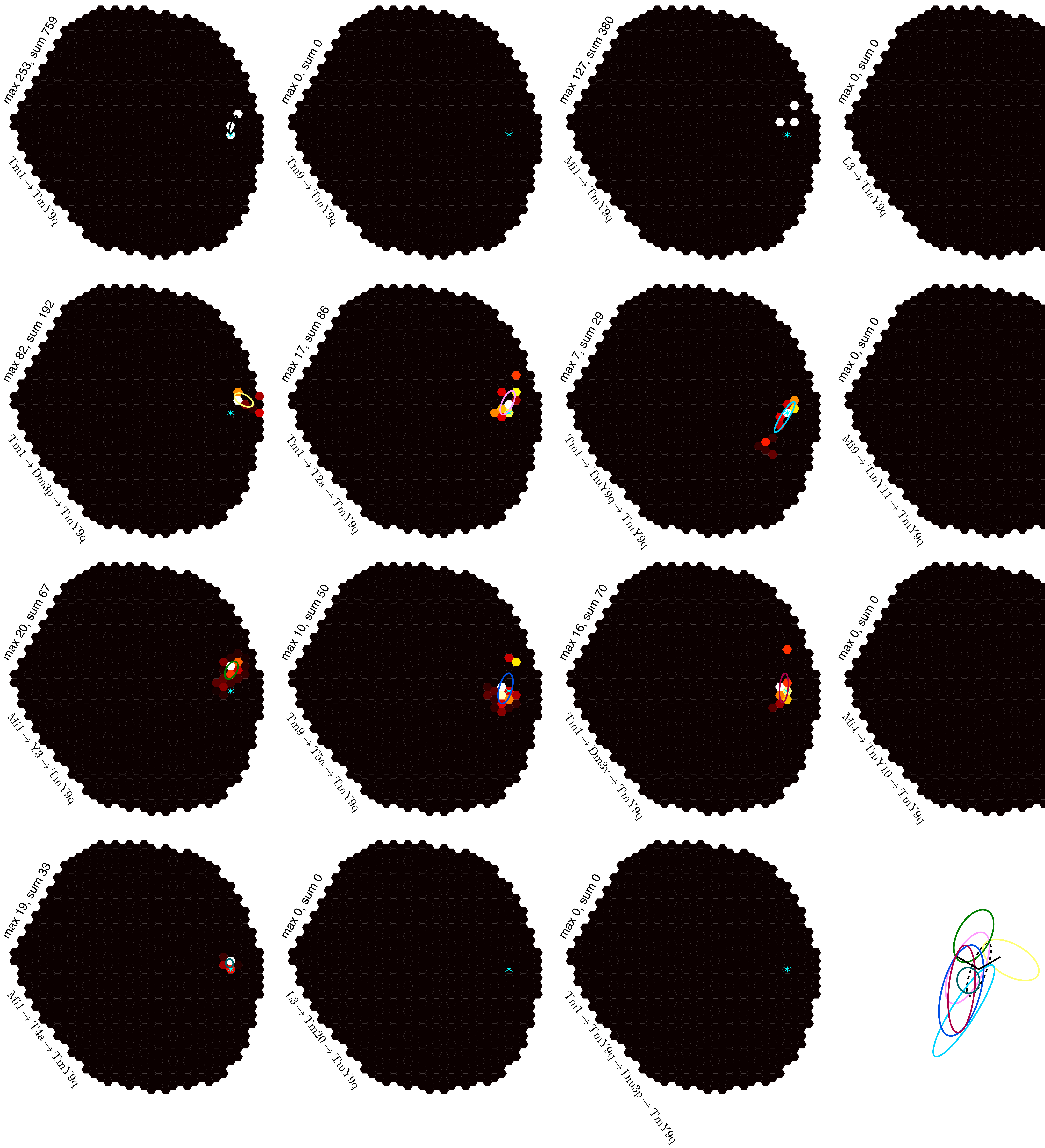
max 253, sum 759
Tm1 → TmY9q
max 0, sum 0
Tm9 → TmY9q
max 127, sum 380
Mi1 → TmY9q
max 0, sum 0
L3 → TmY9q
max 82, sum 192
Tm1 → Dm3p → TmY9q
max 17, sum 86
Tm1 → T2a → TmY9q
max 7, sum 29
Tm1 → TmY9q → TmY9q
max 0, sum 0
Mi9 → TmY11 → TmY9q
max 20, sum 67
Mi1 → Y3 → TmY9q
max 10, sum 50
Tm9 → T5a → TmY9q
max 16, sum 70
Tm1 → Dm3v → TmY9q
max 0, sum 0
Mi4 → TmY10 → TmY9q
max 19, sum 33
Mi1 → T4a → TmY9q
max 0, sum 0
L3 → Tm20 → TmY9q
max 0, sum 0
Tm1 → TmY9q → Dm3p → TmY9q
